# Inappropriate therapy of euvolemic hyponatremia, the most frequent type of hyponatremia in SARS-CoV-2 infection, is associated with increased mortality in COVID-19 patients

**DOI:** 10.3389/fendo.2023.1227059

**Published:** 2023-07-24

**Authors:** Mario Pazos-Guerra, Jorge Gabriel Ruiz-Sánchez, Xavier Pérez-Candel, Celia López-Nevado, Fernando Hernández-Olmeda, Martin Cuesta-Hernández, Javier Martín-Sánchez, Alfonso Luis Calle-Pascual, Isabelle Runkle-de la Vega

**Affiliations:** ^1^ Department of Endocrinology and Nutrition, Instituto de Investigación Sanitaria del Hospital Clínico San Carlos (IdISSC), Hospital Clínico San Carlos, Madrid, Spain; ^2^ Department of Endocrinology and Nutrition, Instituto de Investigación Sanitaria Fundación Jiménez-Díaz (IIS-FJD), Universidad Autónoma de Madrid (UAM), Hospital Universitario Fundación Jiménez Díaz, Madrid, Spain; ^3^ Centro de Investigación Biomédica en Red de Diabetes y Enfermedades Metabólicas Asociadas (CIBERDEM), Madrid, Spain; ^4^ Medicine II Department, Universidad Complutense de Madrid, Madrid, Spain; ^5^ Emergency Department, Instituto de Investigación Sanitaria del Hospital Clínico San Carlos (IdISSC), Emergency Department, Hospital Clínico San Carlos, Madrid, Spain

**Keywords:** hyponatremia, Covid-19, Sars-Cov2, mortality, volemia

## Abstract

**Introduction:**

Admission hyponatremia, frequent in patients hospitalized for COVID-19, has been associated with increased mortality. However, although euvolemic hyponatremia secondary to the Syndrome of Inappropriate Antidiuresis (SIAD) is the single most common cause of hyponatremia in community-acquired pneumonia (CAP), a thorough and rigorous assessment of the volemia of hyponatremic COVID-19 subjects has yet to be described. We sought to identify factors contributing to mortality and hospital length-of-stay (LOS) in hospitalized COVID-19 patients admitted with hyponatremia, taking volemia into account.

**Method:**

Retrospective study of 247 patients admitted with COVID-19 to a tertiary hospital in Madrid, Spain from March 1st through March 30th, 2020, with a glycemia-corrected serum sodium level (SNa) < 135 mmol/L. Variables were collected at admission, at 2nd-3rd day of hospitalization, and ensuing days when hyponatremia persisted. Admission volemia (based on both physical and analytical parameters), therapy, and its adequacy as a function of volemia, were determined.

**Results:**

Age: 68 years [56-81]; 39.9% were female. Median admission SNa was 133 mmol/L [131- 134]. Hyponatremia was mild (SNa 131-134 mmol/L) in 188/247 (76%). Volemia was available in 208/247 patients; 57.2% were euvolemic and the rest (42.8%) hypovolemic. Hyponatremia was left untreated in 154/247 (62.3%) patients. Admission therapy was not concordant with volemia in 43/84 (51.2%). In fact, the majority of treated euvolemic patients received incorrect therapy with isotonic saline (37/41, 90.2%), whereas hypovolemics did not (p=0.001). The latter showed higher mortality rates than those receiving adequate or no therapy (36.7% vs. 19% respectively, p=0.023). The administration of isotonic saline to euvolemic hyponatremic subjects was independently associated with an elevation of in-hospital mortality (Odds Ratio: 3.877, 95%; Confidence Interval: 1.25-12.03).

**Conclusion:**

Hyponatremia in COVID-19 is predominantly euvolemic. Isotonic saline infusion therapy in euvolemic hyponatremic COVID-19 patients can lead to an increased mortality rate. Thus, an exhaustive and precise volemic assessment of the hyponatremic patient with CAP, particularly when due to COVID-19, is mandatory before instauration of therapy, even when hyponatremia is mild.

## 1 Introduction

The severe acute respiratory syndrome caused by coronavirus-2 (SARS-CoV-2), known as Coronavirus disease 2019 (COVID-19), has been affecting populations since the end of 2019. Although the Word Health Organization (WHO) announced on the 5^th^ of May 2023 that COVID-19 was no longer a global public health emergency, this disease is far from being eradicated. The SARS-CoV-2 virus is still capable of inducing infection, severe disease and death, even when optimally treated. In fact, the WHO has reported 1.5 million new cases and 7300 deaths over a 28-day period following the end of the public health emergency, with countries such as Ireland and South Korea communicating an increase in the mortality rate of infected patients (1). Future waves are expected, with unvaccinated populations particularly susceptible, and the protection provided by vaccines waning or less efficient when confronted with new viral variants (2–5). It is therefore important that we review all elements of this infection that are associated with mortality, particularly as regards aspects that can potentially be corrected. One such aspect is hyponatremia.

Hyponatremia in COVID-19 is observed in 20.5% to 35.8% of cases at admission (6, 7). Furthermore, hyponatremia has been detected at some point during hospitalization in 36.9% of COVID-19 patients (8). Although hyponatremia in COVID-19 is habitually mild, hyponatremic patients have been found to be at an increased risk for in-hospital mortality and sepsis (7, 9–11) as well as for sepsis-related death (12). In fact, a recent meta-analysis has found that hyponatremia in COVID-19 is associated with more than double the risk for a poor prognosis, as defined by a composite of mortality, severe COVID-19 and prolonged hospitalization in comparison with eunatremia (13). Although mild hyponatremia has long been considered a marker of underlying disease, it could also be directly related with death in patients with a prior intracellular electrolyte imbalance, such as occurs in heart failure or sepsis (12). However, specific factors associated with mortality in hyponatremic COVID-19 patients have yet to be elucidated. Furthermore, the volemic classification of hyponatremic patients, applying a rigorous approach, is heretofore undescribed.

Hyponatremia has long been recognized in patients with community—acquired pneumonia (CAP). In fact, Cuesta et al. detected hyponatremia (Na ≤130 mmol/L) in 8.3% of patients admitted with CAP in a prospective study conducted in a tertiary Dublin hospital. The Syndrome of Inappropriate Antidiuresis (SIAD) was identified as the most common cause of a low serum Sodium level (SNa) in this study (14). Others have reported rates of hyponatremia in CAP of up to 31.8% (15–17). The microbial agent itself could contribute to the presence of the electrolyte abnormality, as hyponatremia is particularly frequent in patients with *Legionella* pneumonia (18). Pressure on the diaphragm during pneumonia could result in non-osmotic Arginine Vasopressin (AVP) secretion, as what occurs in patients receiving positive pressure ventilation. Furthermore, hypovolemia, that can be seen in patients with gastrointestinal losses, is a potent stimulus of baroreceptor-induced non-osmotic AVP secretion (19–21).

Inflammation itself could be contributing to the development of hyponatremia in COVID-19 patients. Prior to the pandemic, inflammatory cytokines such as Interleukin-6 (IL-6) and Interleukin-1β had been shown to stimulate non-osmotic secretion of AVP (22–25). In patients infected with SARS-CoV-2, Ayus et al. detected that the presence of low SNa on admission was associated with elevated markers of systemic inflammation (26). Ruiz-Sánchez et al. not only observed that patients admitted with hyponatremia had high levels of inflammation markers, they also noted that as the latter descended, hyponatremia improved (27). IL-6, which is often elevated in COVID-19 and related to severity (28), seems to be the pro-inflammatory cytokine most implicated, perhaps because this cytokine often acts in a more endocrine manner than others, acting on target cells at a distance, following transportation through the bloodstream. Berni et al. detected an inverse relationship between levels of SNa and IL-6, in a cohort of COVID-19 patients (6). Frontera et al. ratified these findings (7). These data suggest that inflammation per se plays a role in the hyponatremia of COVID-19.

Before the etiology of a low SNa in a given patient can be identified, the volemia of the subject must be ascertained. Only once the hyponatremic patient has been classified as hypervolemic, hypovolemic, or euvolemic, can a diagnosis be made of the cause of the electrolyte disorder. And not only is a correct volemic classification of hyponatremia necessary for diagnosis, it is also an imperative for correct therapy. In fact, the approaches to COVID-19 hyponatremia that have been published by three scientific societies stress the importance of a correct assessment of volemia in the management of the hyponatremic patient with COVID-19 (29–31). Yet, to date, no rigorous analysis of the volemia of hyponatremic Covid-19 subjects has been reported.

The differentiation of euvolemia from hypovolemia has habitually been considered a pitfall in the approach to the patient with hyponatremia (20, 32). In fact, Chung et al. reported that solely 47% of hypovolemic and 48% of euvolemic patients with hyponatremia were correctly classified (32). The physical examination is the first step in and, indeed, the mainstay of the volemic classification of hyponatremia, with the clinical measurement of the maximum height of the internal jugular vein (IJV) pulse its cornerstone (33). It directly assesses right atrial pressure, and thus intravascular volume (34). Although first described close to a century ago, recent ultrasound studies have validated it as an accurate gauge of right intraatrial pressure (35, 36). A study by Avcil et al. found that its use could be promptly learned by 89% of young physicians and students, with experience only adding to accuracy (36). Additional signs to assess effective circulating volume are the manual ocular pressure (37) and the presence or absence of orthostatic hypotension, the heart rate, and skin turgor (20, 38). The finding of a low urinary sodium value has often been used as an indication of hypovolemia (39). Yet it cannot alone indicate volemic status, as various types of hypovolemic hyponatremia, such as hypoaldosteronism, diuretic use, bicarbonate administration, and cerebral salt-wasting, are characterized by urinary sodium loss. The opposite is also true. A low urinary sodium level does not rule out euvolemic hyponatremia, and can be found in polydipsia, as well as in SIAD patients with a low sodium intake (33, 40–42).

Heretofore no study has applied a rigorous approach to the assertation of volemia in hyponatremic COVID-19 patients, based on the physical examination, and supported by laboratory findings. Nor has the response to therapy as a function of volemic status been investigated. Yet, as previously stated, a correct evaluation of volemia is essential to assure the hyponatremic patient is properly treated. The purpose of this study is to determine the rates of euvolemic, hypovolemic and hypervolemic hyponatremia in patients admitted with COVID-19 and analyze whether admission volemia and volemia-guided therapies are associated with in-hospital mortality, hospital length of stay (LOS), and the evolution of hyponatremic patients. We further sought to detect the prevalence and mortality rates in hyponatremic COVID-19 patients, and the factors related to the latter.

## 2 Materials and methods

All adult (≥ 18 years of age) patients with confirmed COVID-19 and hyponatremia at admission, hospitalized at the Hospital Clínico San Carlos (HCSC) of Madrid, Spain, during the first wave of the COVID-19 pandemic in March of 2020, were analyzed. A total of 750 COVID-19 patients were attended during this period, and their clinical records were reviewed. Only patients with a positive reverse-transcriptase polymerase chain reaction test for SARS-COV2 were included. Hyponatremia was defined as a serum sodium level (SNa) < 135 mmol/L after correction for glycemia when appropriate (43). This study complied with the ethical principles of the Declaration of Helsinki and Good Clinical Practice Guidelines and was approved by the Research Ethics Committee of the HCSC (20/396-E_COVID). Written informed consent was waived because of the health alarm situation and the retrospective design.

The following variables were registered: age, sex, previously diagnosed comorbidities [hypertension, diabetes mellitus (DM), chronic obstructive pulmonary disease (COPD), chronic kidney disease (CKD), cardiovascular events (CV) such as peripheral arterial disease, ictus or ischemic cardiomyopathy, and a history of an active oncological disease], chronic medication [angiotensin-converting enzyme inhibitors (ACEs), angiotensin-2 receptor antagonists (ARBs), mineralocorticoid receptor blockers (MRBs), diuretics, selective serotonin reuptake inhibitors (SSRIs), non-SSRI antidepressants and antiepileptics (carbamazepine, oxcarbazepine, or eslicarbazepine, valproate and levetiracetam)]. Clinical parameters were collected at admission, the 2nd-3rd day of hospitalization, and at the patient’s discharge [oxygen saturation (OSat), SNa, glycemia, serum creatinine (SC), glomerular filtration rate (GFR) with CKD-EPI formula, serum potassium (SK), Serum urea (SU), total serum proteins (TP), serum albumin (SAlb)]. Additionally, information on admission treatment for hyponatremia [none, fluid restriction (FR), isotonic saline infusion (ISI), furosemide, oral NaCl tablets, tolvaptan, or urea] and admissions volemic status was collected. In patients with persistent hyponatremia, SNa levels from additional days were registered, permitting the calculation of the total number of days on which SNa was measured, and days with hyponatremia during hospitalization.

Volemia was categorized as hypovolemia, euvolemia or hypervolemia. Hypovolemia, based on the data recorded in the clinical records, was defined as a maximum height of the internal jugular pulse (HIJP) below 1 cm over the sternal angle with the patient reclined at 0-30°, plus at least two of the following: thirst, orthostatic symptoms, blood pressure ≤ 90/60 mmHg, heart rate ≥ 90 bpm, urinary sodium ≤ 30 mmol/L in the absence of polydipsia or a low sodium intake, and/or a rise in SC and/or SU accompanying the descent in SNa (40, 44, 45). If HIJP was not available, hypovolemia was determined by the presence of at least three of the aforementioned clinical features. Euvolemia was defined as the absence of symptoms/signs of hypovolemia and hypervolemia, with a HIJP at 1-3 cm above the sternal angle. Hypervolemia was diagnosed when clinical signs of heart failure or liver cirrhosis were present, in the former case coinciding with HIJP > 3 cm above the sternal angle when available.

Adequacy of treatment was categorized as “adequate” or “inadequate” as follows: “Adequate” when euvolemic hyponatremia patients had received therapy with FR, furosemide, urea, or tolvaptan, as well as when hypovolemic patients were administered ISI. Therapy was considered “Inadequate” when euvolemic hyponatremic patients received ISI, and when hypovolemic hyponatremic patients received furosemide, urea, tolvaptan, or if FR was indicated. The administration of NaCl tablets as sole therapy was considered inadequate in all cases, as isolated salt administration per se is not a therapy for hyponatremia (46).

### 2.1 Statistical analysis

Categorical variables are described in frequencies and percentages, and quantitative variables in median and interquartile range [IQR] when non-parametric or means and standard deviation (±) when parametric. The comparative analysis was performed with the Chi-squared or Fisher test for categorical variables, and with the Mann-Whitney U or Kruskal-Wallis tests (if non-parametric) and T-student or ANOVA tests (if parametric) for quantitative variables.

Multivariate logistic and linear regression analyses were performed. The logistic model of the multivariable analysis included all variables with either a p significance < 0.1 in the univariate analysis or with clinical relevance. Forward steps methodology was used in both multivariate analyses. A two-tailed p value < 0.05 was considered statistically significant. Ninety-five percent confidence intervals (95% CI) were calculated for determination of the Odds Ratio (OR) and coefficient B (B). Statistical analysis was performed using SPSS version 25 (IBM Corp., N.Y.).

## 3 Results

Two hundred forty-seven (32.9%) of the 750 Covid-19 patients admitted presented with hyponatremia and were included in the current study. The median age was 68 years [56-81], and 99/247 (39.9%) were female. An admission SNa ranging from 131-134 mmol/L was found in 188/247 (76%) patients, a SNa ≤130 mmol/L in 52 (21.1%), with SNa ≤125 mmol/L detected in only 7 (2.8%). In our cohort, 53 (21.5%) patients died during hospitalization. Baseline characteristics and admission factors associated with hospital mortality in the studied cohort are displayed in **Table 1**. A detailed analysis of mortality in our cohort is described below.

**Table 1:** Baseline characteristics of patients at admission compared as a function of in-hospital mortality or survival.

	Total	Died	Survived	*p*
	N= 247	53 (21.5%)	194 (78.5%)	
*Age, years*	68 [56-81]	80 [73-86]	65 [53-76.3]	<0.001*
Females, n (%)	98 (39.7)	25 (47.2)	73 (37.6)	0.208
Comorbidities				
*Diabetes Mellitus, n (%)*	53 (21.5)	18 (34)	35 (18)	0.012*
*Hypertension, n (%)*	131 (53)	37 (69.8)	94 (48.5)	0.006*
COPD, n (%)	24 (9.7)	7 (2.8)	17 (6.9)	0.333
*CKD, n (%)*	26 (10.5)	11 (20.8)	15 (7.7)	0.006*
*Oncological, n (%)*	15/59 (25.4)	5/8 (62.5)	10/51 (19.6)	0.020*
CV events, n (%)	43 (17.4)	12 (22.6)	31 (16)	0.257
Previous chronic medication				
ACEi, n (%)	54 (21.9)	14 (26.4)	40 (20.6)	0.366
ARB, n (%)	48 (19.4)	15 (28.3)	33 (17)	0.066
MRB, n (%)	11 (4.5)	3 (5.7)	8 (4.1)	0.703
*Thiazide/thiazide-like, n (%)*	50 (20.2)	16 (30.2)	34 (17.5)	0.042*
Loop diuretic, n (%)	19 (7.7)	6 (11.3)	13 (6.7)	0.263
SSRI, n (%)	19 (7.7)	6 (11.3)	13 (6.7)	0.263
Other antidepressants, n (%)	8 (3.2)	4 (7.5)	4 (2.1)	0.067
Antiepileptics, n (%)	7 (2.8)	2 (3.8)	5 (2.6)	0.645
Clinical data at admission				
Volemic status				0.502
Hypovolemia, n (%)	89/208 (42.8)	23/49 (46.9)	66/159 (41.5)	
Euvolemia, n (%)	119/208 (57.2)	26/49 (53.1)	93/159 (58.5)	
Hypervolemia, n (%)	0	0	0	
*Oxygen saturation*	94 [90-96]	90 [84-93]	94 [91-97]	<0.001*
* OSat<95% rate, n (%)*	139/236 (58.9)	43/50 (86)	96/186 (51.6)	<0.001*
Serum Sodium, mmol/L	133 [131-134]	133 [132-133.5]	133 [131-134]	0.964
Serum Potassium, mmol/L	4.1 [3.9-4.5]	4.2 [4-4.8]	4.1 [3.8-4.4]	0.127
Glycemia, mg/dL	117 [105-132]	122 [109-149]	115 [104-130]	0.245
*Serum Creatinine, mg/dL*	0.93 [0.71-1.15]	1.15 [0.85-1.79]	0.87 [0.69-1.05]	0.011*
*Glomerular filtration rate, ml/min (CKD-EPI)*	81 [58-96]	50 [32-83]	85 [65-100]	<0.001*
Adequate Volemia-based treatment	39/84 (46.4%)	12/26 (42.9%)	27/56 (48.2%)	0.643

COPD, Chronic obstructive pulmonary disease; CKD, Chronic kidney disease; CV, cardiovascular; ACEi, Angiotensin converting-enzyme inhibitor; ARB, Angiotensin-2 receptor blockers; MRB, mineralocorticoid receptor blockers; SSRI, selective serotonin reuptake inhibitors.

*Significant results in italics (p<0.05).

### 3.1 Volemic status

Volemic status data was available in 208 patients, 89 of whom (42.8%) were classified as hypovolemic and 119/208 (57.2%) as euvolemic. No patient was found to be hypervolemic. The factors associated with hypovolemia or euvolemia at admission are described in **Table 2**. Specific data regarding gastrointestinal symptoms as a potential cause of hypovolemia were not registered. In no patient was the presence of hemorrhage detected.

**Table 2:** Characteristics at admission of hypovolemic and euvolemic patients.

	hypovolemic	euvolemic	*p*
	89 (42.8%)	119 (57.2%)	
Age, years	72 [62-83]	68 [57-81]	0.262
Females, n (%)	38 (42.7)	49 (41.2)	0.826
Comorbidities			
Diabetes Mellitus, n (%)	22 (24.7)	27 (22.7)	0.733
*Hypertension, n (%)*	65 (73)	56 (47.1)	<0.001*
COPD, n (%)	10 (11.2)	14 (11.8)	0.906
CKD, n (%)	11 (12.4)	14 (11.8)	0.896
Oncological, n (%)	10/34 (29.4)	5/21 (23.8)	0.650
CV events, n (%)	16 (18)	26 (21.8)	0.491
Previous chronic medication			
*Use of any drug, n (%)*	59 (66.3)	56 (47.1)	0.006*
*Use of Anti-RAAS drugs, n (%)*	46 (51.7)	38 (31.9)	0.004*
ACEi, n (%)	24 (27)	25 (21)	0.316
*ARB, n (%)*	26 (29.2)	17 (14.3)	0.009*
MRB, n (%)	5 (5.6)	6 (5)	0.854
Diuretics, n (%)	30 (33.7)	34 (28.6)	0.427
Thiazides, n (%)	25 (28.1)	22 (18.5)	0.102
Loop diuretics, n (%)	6 (6.7)	13 (10.4)	0.300
SSRI, n (%)	5 (5.6)	3 (2.5)	0.292
Other antidepressant, n (%)	4 (7.5)	4 (2.1)	0.067
Antiepileptics, n (%)	5 (5.6)	1 (0.8)	0.086
Clinical data at admission			
Oxygen saturation, %	93 [89-96]	94 [90-96]	0.480
Serum Sodium, mmol/L	132 [130-133]	133 [132-134]	0.253
Serum Potassium, mmol/L	4.1 [3.8-4.5]	4.1 [3.9-4.5]	0.869
Glycemia, mg/dL	118 [107-136]	115 [103-132]	0.400
*Serum Creatinine, mg/dL*	1.06 [0.89-1.41]	0.83 [0.66-1]	<0.001*
*GFR, ml/min (CKD-EPI)*	61 [42-83]	86 [71-99]	<0.001*
*Serum Urea, mg/dL*	53 [33-71]	33 [25-45]	<0.001*

COPD, Chronic obstructive pulmonary disease; CKD, Chronic kidney disease; CV, cardiovascular; ACEi, Angiotensin converting-enzyme inhibitor; ARB, Angiotensin-2 receptor blockers; MRB, mineralocorticoid receptor blockers, SSRI, selective serotonin reuptake inhibitors; GFR, glomerular filtration rate.

*Significant results in italics (p<0.05).

Patients with hypovolemia were more likely to present an admission SNa ≤ 130 mmol/L than those euvolemic (33.7% vs. 16% respectively, p= 0.003), while similar rates of a SNa ≤ 125 mmol/L were found in hypovolemia and euvolemia (3.4% vs. 2.5% respectively, p=1).

We did not find any impact of admission volemic status on mortality, LOS, persistence of hyponatremia at 2nd-3rd day, nor on the number of days of hospitalization with hyponatremia (**Table 3**). SNa values at the 2nd-3rd day of hospitalization were available in 180 of the patients in whom volemic data were registered. SNa was measured during hospitalization a median of 6 days [3.5-10] in hypovolemic and 5 days [4-10] in euvolemic patients, with SNa values determined in 58% [45-71] and 50% [40-68] (p= 0.230) of the days of hospital stay of these patients respectively. The median nadir SNa during hospitalization was 132 mmol/L [129-133] in hypovolemic and 133 mmol/L [131-134] in euvolemic subjects (p= 0.158).

**Table 3:** Impact of admission volemia on clinical outcomes during hospitalization.

	Hypovolemia	Euvolemia	*p*
Mortality rate, n (%)	21 (24.0%)	25 (21.3%)	0.502
Length of hospital stay, days	14.3 +/-13.4	15.4 +/-12.5	0.465
Hyponatremia at 2^nd^-3^rd^ day, n(%)	26 (32.9)	35 (34.7)	0.806
Days with hyponatremia	2 [1-3]	1 [1-3]	0.547

### 3.2 Admission therapy of hyponatremia

No therapy for hyponatremia was ordered in a majority of patients: 154/247 (62.3%). 74/247 (30%) patients were treated with ISI, 4/247 (1.6%) with furosemide plus NaCl tablets, and 11/247 (4.5%) solely with NaCl tablets. Chronic, rather than acute treatment with a loop diuretic, was not considered to be a treatment of hyponatremia (44). No patient received tolvaptan nor urea. When therapy or the lack of same was evaluated according to volemia, 46/89 (51.7%) of hypovolemic patients did not receive any treatment for hyponatremia, whereas 78/119 (65%) of euvolemic hyponatremic patients received no treatment. Specific therapy according to volemia is presented in **Table 4**.

**Table 4:** Treatment according to volemia.

	Hypovolemia n=89	Euvolemia n=119
No therapy ordered	46 (51.7%)	78 (65.6%)
Isotonic saline infusion	37 (41.6%)	30 (25.2%)*
Fluid Restriction	2 (2.2%)*	2 (1.7%)
Furosemide+ NaCl tablets	2 (2.2%)*	2 (1.7%)
NaCl tablets	2 (2.2%)*	7 (5.8%)*
Total number of cases with inappropriate therapy	*6 (6.6%)*	*37 (31.1%)*

Results given as number of patients (%). *Volemia-inappropriate therapy.

When identifying patients who received specific therapy for hyponatremia in whom volemic status had been ascertained, 84 subjects remained (43 hypovolemic and 41 euvolemic). In these, we analyzed the adequacy of their treatment as a function of volemic status. Therapy was not in accordance with volemic status in a total of 43/84 (51.2%) of treated subjects. The treatment ordered was more frequently incorrect for volemic status in euvolemics, with 37/41 (90.2%) administered an inadequate treatment, whereas only 6/43 (14%) hypovolemics were incorrectly treated. The difference between the two groups was statistically significant (p<0.001).

### 3.3 Mortality in hyponatremia and impact of treatment on mortality

Factors associated with in-hospital mortality in the univariate analysis are displayed in **Table 1**. Neither an admission SNa ≤ 130 mmol/L (p=0.412), nor a SNa ≤ 125 mmol/L (p= 0.645) were associated with mortality.

The mortality rate was similar when comparing those adequately and inadequately treated (31.7% vs. 34.9%, p= 0.758). Yet, analysis of the entire cohort detected a higher mortality rate in those treated when compared with those not receiving therapy (32.3% vs. 14.9%, p= 0.001).

**Figure 1:**
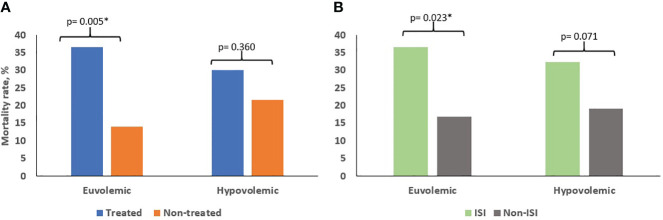
Mortality rates according to the presence **(A)** or type **(B)** of treatment received, compared by volemic status. In patients with euvolemic hyponatremia, the presence of any hyponatremia treatment, as compared with its absence, was significatively associated with higher mortality rates. However, this was not the case in subjects with hypovolemic hyponatremia. Subanalysis demonstrated that ISI therapy in euvolemic hyponatremic patients was associated with a higher mortality. ISI: isotonic saline infusion *p<0.05.

Upon further analysis, when classifying patients by volemic status, the mortality rate of treated euvolemic subjects was significantly higher than that of euvolemics not receiving therapy (36.6% vs. 14.1% respectively, p= 0.005), whereas this difference was not observed in the case of hypovolemics (30.2% vs. 21.7% respectively, p= 0.360) (**Figure 1A**), nor when comparing those treated with ISI with subjects not receiving ISI irrespective of volemia (32.4% vs. 21.2% respectively, p= 0.231). Conducting an in-depth analysis, we detected that the mortality rate of patients with euvolemic hyponatremia incorrectly treated (in all cases, with ISI) was significantly higher than in those receiving adequate or no therapy (36.7% vs. 19% respectively, p= 0.023). This was not the case in hypovolemics when an adequate treatment (in all cases, with ISI) was compared to an incorrect or absent treatment (32.4% vs. 19.2% respectively, p = 0.071) (**Figure 1B**). Thus, therapy with isotonic saline was associated with an increase in the mortality rate of patients with euvolemic hyponatremia.

We performed a first multivariate analysis that included age, sex, and the following admission variables: oncological history, CKD, DM, hypertension, previous thiazide treatment, OSat at admission, SC at admission in the presence of any treatment for hyponatremia. The OSat levels at admission (OR: 0.88, 95%CI 0.78 to 0.99), and an oncological history (OR: 11.9, 95%CI 1.69 to 83.6) were independently associated with mortality. Given that the registry of an oncological history was available only in the 23.8% of the cohort, thereby affecting the potency of the analysis, we excluded this variable in the following model. Thus, in the second multivariate analysis, age (OR: 1.066, 95%CI: 1.03-1.1), OSat levels (OR: 0.872, 95%CI: 0.82-0.93), SC levels (OR: 1.35, 95%CI: 1.09-1.67) and the presence of treatment for hyponatremia (OR: 2.257. 95%CI: 1.06-4.81) were independently associated with mortality. However, when in a third analysis, we replaced the presence of any treatment of hyponatremia with the adequacy of therapy, only age (OR: 1.054, 95%CI: 1.01-1.1) and OSat levels (OR: 0.869, 95%CI: 0.79-0.96) remained associated with mortality in the entire cohort.

We performed another multivariate analysis (adjusting by age, sex, CKD, DM, hypertension, previous thiazide treatment, admission OSat, SC and therapy with ISI) assessing the effect of therapy as a function of volemic status. We found that the incorrect therapy of euvolemic subjects with ISI to be independently associated with mortality (OR: 3.877. 95%CI: 1.25-12.03). In hypovolemic subjects, this was not the case.

**Figure 2:**
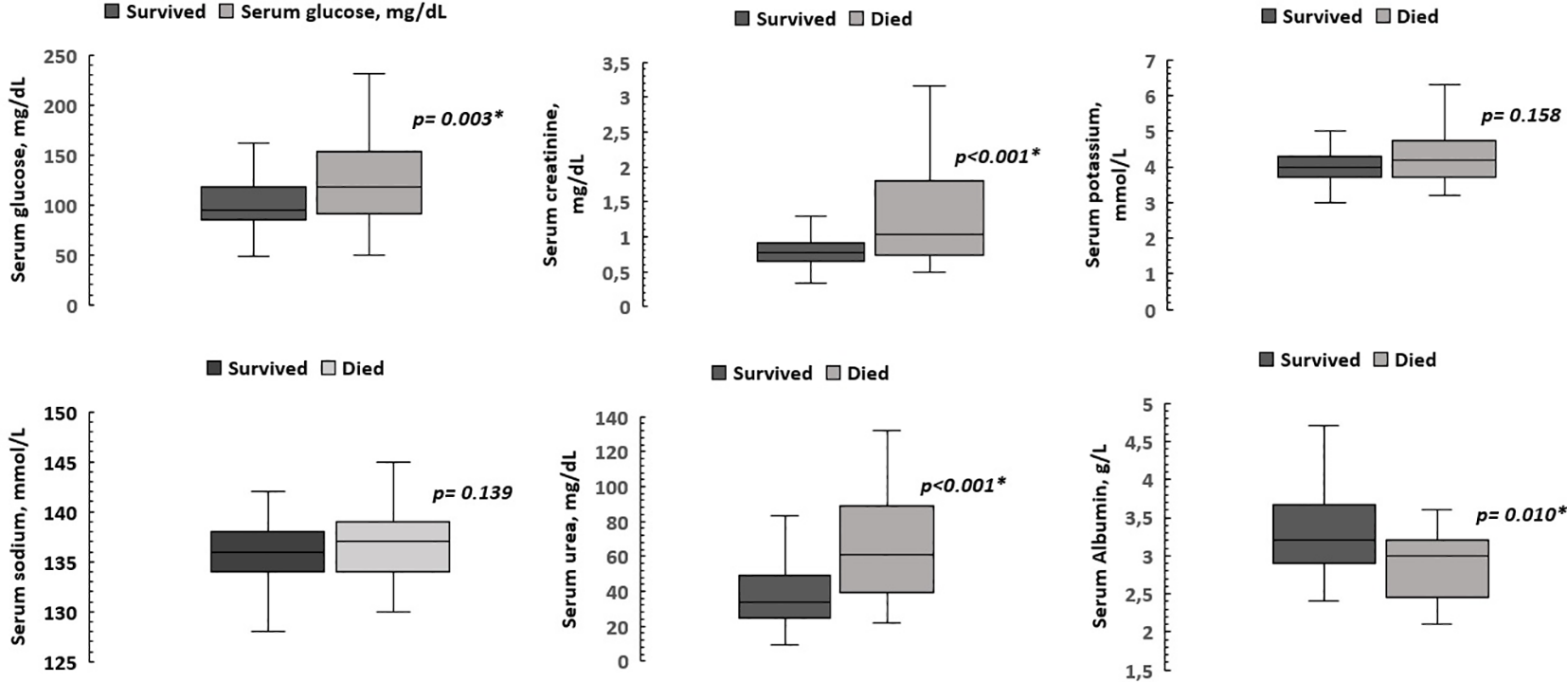
Comparison of biochemical parameters at the 2^nd^-3^rd^ hospitalization day of patients who died and those who survived. Patients who died had higher medians of glycemia, serum creatinine and serum urea, as well as a lower median serum albumin level. The median serum sodium and potassium levels were similar. *p < 0.05.

Additionally, we compared the medians of SNa, glycemia, SC, SK, urea, and SAlb at the 2nd-3rd hospitalization day of the patients who died and those who survived (**Figure 2**). No differences were found in the medians of SNa at the 2nd-3rd hospitalization day of both groups. Furthermore, neither the persistence of hyponatremia (p=0.594) nor the decrement of SNa at 2nd-3rd day of hospitalization were associated with mortality (p= 0.603).

However, we found that the patients who died had a higher median rate of hospitalization days with hyponatremia (25% [15-45] vs. 17% [9-33], p=0.007), as well as a lower median LOS than survivors (7 [4-12] vs. 12 [7-20] days, p <0.001). Thus, in patients who died, there was also a higher median rate of SNa measurements with hyponatremia (50% [33-100] vs. 33% [20-60], p= 0.008). A univariate logistic regression showed that the higher the rate of hospitalization days with hyponatremia, the higher the risk for mortality (OR: 3.583, 95%CI: 1.25-10.27).

### 3.4 Impact of treatment on SNa during hospitalization and LOS

SNa values at the 2nd-3rd day of hospitalization were available in 210 patients of the entire cohort, and a mean increment in SNa of 4 ± 3.2 mmol/L was observed. SNa decreased at the 2nd-3rd day of hospitalization in 16 (7.6%). Hyponatremia persisted in 72/210 (34.3%) patients. Initial admission treatment or non-treatment of hyponatremia did not modify the rate of patients showing a SNa descent (3.5% vs. 10.4% respectively, p= 0.109), nor did the presence or absence of inadequate therapy (2.9% vs. 4.8% respectively, p =1), upon analyzing the entire cohort.

Although the difference were not statistically significant, we did observe in the entire cohort that patients adequately treated were less prone to remained hyponatremic at the 2nd-3rd day than those receiving inadequate therapy (32.4% vs. 40% respectively, p=0.490) and had a slightly higher median SNa (137 mmol/L [133-138] vs. 135 mmol/L [133-138] respectively, p= 0.219) as well as a higher increment of SNa levels (+5 mmol/L [3-8] vs. +4 mmol/L [2-7] respectively, p= 0.854) at 2nd-3rd day than those incorrectly treated. These differences disappeared upon analyzing euvolemic and hypovolemic subjects independently.

The median LOS (12 [7-20] vs. 11 [6-22] days, p= 0.831), days with hyponatremia during hospitalization (2 [1-5] vs. 2 [1-3] days, p= 0.938), and the mean nadir SNa values (130.6 ± 2.9 vs. 131.1 ± 2.8 mmol/L, p= 0.367) were similar when comparing adequately and inadequately treated patients.

## 4 Discussion

Close to a third (32.9%) of the patients hospitalized for COVID-19 during the first wave presented with hyponatremia at admission in the current study. Their mean age was 68, and they were predominantly male (over 60%). In the vast majority (76%), hyponatremia was mild, with glycemia-corrected SNa levels of 131-134 mmol/L. A SNa ≤125 mmol/L was found in only 2.8% of the patients studied. These findings are in accordance with prior studies of COVID-19 patients (6, 7). Thus, subjects hospitalized with COVID-19 during the first-wave frequently presented with hyponatremia, albeit mild, when first attended at the emergency room (ER).

We found that a majority of the hyponatremic patients at admission were euvolemic (57.2%), and the remaining subjects hypovolemic (42.8%). No hyponatremic patient was hypervolemic. To our knowledge, this report is the first to describe the volemia of hyponatremic COVID-19 patients with a rigorous approach that includes the physical examination, as well as biochemical data, and the evolution of the latter during hospitalization.

The volemic classification of patients with hyponatremia remains a challenge for clinicians, and error is frequent (20). One reason for this failure could be the progressive loss of the use of the physical examination in clinical medicine, such as the inspection of the maximum height of the internal jugular pulse as an accurate gauge of right intra-atrial pressure (34). Another reason for confusion is the consideration of a low urinary sodium as a firm indication of hypovolemia, with a normal or high level defining euvolemia. Yet, as we have stated above, this is far from true, and overlap between urinary sodium levels of euvolemic and hypovolemic hyponatremic subjects is considerable (33, 39, 42). Thus, volemic classification cannot be based on an indirect variable such as urinary sodium alone (40, 41).

Of greater use in the differential diagnosis of hypovolemic from euvolemic hyponatremia, among indirect measurements, could be the evaluation of the changes in serum creatinine levels in relation to modifications in serum sodium levels, since in hypovolemia, creatinine levels rise as SNa descends. This method, long used in clinical settings, has been recently validated by Ruiz- Sanchez et al, although its generalized use warrants further investigation (40, 44, 45). Although some studies have proposed that SIAD could be the most frequent cause of hyponatremia in COVID-19 (47, 48), as has a recent meta-analysis (49), none of them have included the specific volemic evaluation necessary for this diagnosis.

To our knowledge, only 3 reports have attempted to classify volemia in COVID-19 hyponatremia, all exclusively using one or more of the aforementioned biochemical parameters as guides (8, 27, 50). Tzoulis et al. based volemic classification on indirect parameters such as a urinary Na level <30 mmol/L and/or a serum urea (SU) level >5 mmol/L. With this methodology, they found the vast majority of patients to be hypovolemic (75%). They further detected a higher mortality rate in hypovolemic patients when compared to euvolemics (8).

The other 2 studies (27, 50) used the evolution of SC levels from hyponatremia to eunatremia as a way to estimate volemia in patients. Ruiz Sanchez et al. also took other symptoms/signs into account such as thirst, orthostatic symptoms, blood pressure, heart rate and urinary sodium. Both studies found a high rate of hypovolemic patients, 53.9% and 55.1% respectively, higher than our 42.8%, albeit lower than the 75% reported by Tzoulis et al. (8). We believe the discrepancy in results can be attributed to the fact that the current study uses a comprehensive approach to volemic classification, heretofore undescribed in the study of COVID-19 hyponatremia.

Our results in terms of the predominance of euvolemia, however, are in line with those of Cuesta et al., in a prospective study with a rigorous approach to volemic classification, in patients with non-COVID-19 CAP (14). The aforementioned authors found that 46% of their patients had SIAD-induced euvolemic hyponatremia, 42% hypovolemic hyponatremia, and 12% hypervolemic hyponatremia. A further 6% had euvolemic hyponatremia caused by secondary adrenal insufficiency. Thus, excluding hypervolemic hyponatremia, 54% of patients were euvolemic. In contrast with the findings of Cuesta et al., we did not detect a single case of hypervolemic hyponatremia. Nor have prior reports of hyponatremic COVID-19 patients described patients with hypervolemia (49), in spite of the fact that the latter is more easily distinguished than euvolemia and hypovolemia. The absence of hypervolemic patients could possibly be related with the frequent presence of gastrointestinal losses in COVID-19, together with a reduced fluid intake related to ageusia, both factors making fluid overload less likely.

Although an initial analysis detected that patients who received any treatment for hyponatremia died at a higher rate than those who did not, further analysis indicated that this finding was due to the high rate of administration of inadequate therapy: a full 51.2% of patients did not receive treatment in accordance with their volemia. In fact, error was practically a constant in the therapy of euvolemics, 90% of whom were incorrectly treated, specifically with isotonic saline infusion (ISI). Thus, an increased mortality rate was observed in treated euvolemics versus those euvolemics not receiving any therapy at all. Of greater importance was the finding that the patients with euvolemic hyponatremia at admission who had received therapy with ISI had a significantly higher mortality rate than those receiving no treatment whatsoever. In fact, ISI therapy was independently associated in euvolemics with an OR for a fatal outcome of 3.87. This is in contrast with the use of ISI in patients with hypovolemic hyponatremia, in whom ISI is indicated, and its administration did not induce a negative response. Thus, the finding that therapy was associated with a worse outcome should not be misconstrued as an attestation that hyponatremia should not be treated. Rather, it illustrates the importance of the administration of a correct therapy. And for the latter to be possible, an accurate volemic classification of the hyponatremic patient is essential.

In the previously cited article by Cuesta et al. the authors, in concordance with our results, detected no significant differences in the mortality rates of euvolemic versus hypovolemic subjects with hyponatremia and CAP (14). Nor did the aforementioned study by Ruiz et al. in COVID- 19 patients detect volemia-based differences in mortality (27). Chan et al. did find euvolemic hyponatremia at admission to be predictive of a higher 90-day mortality (HR 2.03 95%CI 1.77-2.32, p<0.01) (50). However, none of these studies evaluated the evolution of patients as a function of treatment for hyponatremia. In fact, only one study, to our knowledge, described the impact of the correction of hyponatremia on prognosis of COVID-19 patients, demonstrating that a SNa improvement at 72-96 hours after admission was associated with a higher survival rate (51). Neither volemia nor type of therapy were assessed.

Isotonic saline should not be administered to patients with euvolemic hyponatremia. It may not only fail to correct hyponatremia, but can even worsen it (40, 52) by inducing an increase in total body water in patients with antidiuresis (53, 54). Yet therapeutic errors in the management of hyponatremic patients are frequent, particularly in the case of euvolemic subjects, albeit not to the extent observed in the current study. Berkamn et al. found that the postulated clinical diagnosis was considered accurate in only 37.5% of hyponatremic patients (52). And prior studies have reported the frequent misuse of ISI therapy in patients with euvolemic hyponatremia (55–57). In fact, the Hyponatremia Registry study indicated that SIAD, the most common etiology of euvolemic hyponatremia, was incorrectly treated in 66% of cases (58).

Treatment errors in the management of hyponatremia have been associated with a higher mortality rate (59), but in the context of marked hyponatremia, with SNa ≤ 125 mmol/L, in contrast with the cases we have reported herein. We believe this to be the first study to find a reduced survival rate directly related to the inappropriate use of isotonic saline in subjects with euvolemic hyponatremia. The finding is of particular relevance given that approximately 97% of cases presented mild or moderate hyponatremia.

The high rate of incorrect therapy observed in the current study could be related to the specialties of the attending physicians during the initial COVID-19 crisis, that included surgeons, and ophthalmologists, many of whom had no prior experience in emergency medicine. Additionally, the extraordinary burden placed on Emergency Room personnel during the first wave of COVID-19 could also have had an impact on the assessment of hyponatremia, particularly when mild, and scant attention paid to it. In any event, our findings highlight the importance of a correct volemic assessment in the approach to the patient with hyponatremia, as incorrect therapy was related with fatal outcomes.

The precise mechanism by which the use of isotonic saline in the euvolemic hyponatremic patients we have studied was associated with a higher mortality rate is unclear. Although we did detect higher rates of persistence or worsening of hyponatremia, and lower levels of SNa at the 2nd-3rd day of hospitalization in patients treated inadequately, these differences did not attain statistical significance. The latter could perhaps be related to the fact that the rates of moderate and severe hyponatremia were low, and mild hyponatremia predominant. Another possible contributing factor to the negative outcome observed in ISI–treated euvolemic patients could have been the development of fluid overload, although pertinent clinical information was not registered.

We detected a mortality rate of 21.5% in the entire group of COVID-19 patients. Our finding is within the range that has been described by other authors, from 20 to 30·% (6, 8–13, 49, 51, 60). Although the presence of hyponatremia is clearly related with reduced survival (6, 9–13, 60), risk factors for mortality in hospitalized COVID-19 patients with hyponatremia have been sought in only in two studies to date. In one, admission SNa values of hyponatremic patients were independently correlated with the hospital mortality risk (Hazard ratio [HR]: 0.53, 95% CI: 0.32 to 0.87). However, this association was lost upon including SC in a second multivariable model (27). The other study correlated moderate and severe hyponatremia as well as euvolemic hyponatremia with a higher mortality rate (50), but the elevated number of patients with moderate or severe hyponatremia analyzed by the authors makes comparison with our cohort difficult. In the current study, we found that the main admission factors associated with mortality were age, OSat levels, SC levels, and therapy for hyponatremia. When a history of oncological disease was present, it too was a significant risk factor.

We detected no relationship between mortality and admission SNa levels per se in the current study, nor with admission volemia, nor with the persistence of hyponatremia at the 2nd–3rd day of hospitalization. However, the rates in normonatremic subjects with COVID-19 were not studied, and thus differences attributable to the presence or absence of hyponatremia itself could not be evaluated. The adequacy of treatment in the whole cohort and in hypovolemic patients had no impact on mortality. Sustained hyponatremia during hospitalization, on the other hand, was associated with a higher mortality rate, as patients with a higher rate of days of hospitalization with hyponatremia showed an increased risk for in-hospital death (OR 3.583). Similar findings have been described in non-COVID-19 patients receiving parenteral nutrition (61). Thus, the initial degree of hyponatremia in and of itself did not influence the mortality rate. However, persistence of hyponatremia did.

Our study has several important limitations. It is a retrospective study, and therefore, some collection biases could have occurred. Data on normonatremic subjects was not available to compare with those hyponatremic, making the identification of a possible link of hyponatremia per se to mortality impossible. Furthermore, the management of patients could not be considered to be standard clinical practice, as patients were attended during the first wave of the pandemic in the setting of a public health crisis, in which the health system was overwhelmed. In fact, multivariable analyses showed an association of these variables with mortality Another important weakness is the lack of information regarding previous episodes of hyponatremia, as well as symptoms such as vomit, nausea or diarrhea, or other comorbidities at admission that can be a cause per se of hyponatremia. Another serious limitation of the study is that these data were not released in a timely manner, soon after the start of the pandemic and its first wave. However, given the continued presence of COVID-19 infection and the possible relevance of the results to the therapy of euvolemic hyponatremia in general, upon analysis of the data initially collected, we feel that the results have enough clinical relevance to merit their communication to the scientific community.

The main strength of our study is the meticulous collection of volemic data and the assessment of the adequacy of the treatment of hyponatremia. Furthermore, all patients of the tertiary HCSC admitted during the time of study were included, permitting a sufficient number of subjects for statistical analysis. Another advantage is the homogeneity of the patients studied as regards the microbial origin of their CAP, as well as the setting in which they were hospitalized, perhaps facilitating the detection of the consequences of inappropriate therapy for hyponatremia.

In conclusion, the majority of COVID-19 patients with hyponatremia are euvolemic, and the descent in serum sodium is mild. Furthermore, in most cases, eunatremia will be attained by the 2nd-3rd day on the wards, regardless of therapy. However, those patients who are euvolemic and receive iv isotonic saline infusion are at a higher risk for in-hospital mortality, as are hyponatremic patients of advanced age, those presenting with a reduced OSat, an increased SC, and/or an oncological history. Although COVID-19 is no longer a public health emergency, it is still a prevalent disease that will presumably remain with us for many years (2, 62). We thus recommend the performance of an exhaustive and precise volemic assessment before initiation of therapy in COVID-19 cases with mild-moderate hyponatremia, as well as in other causes of CAP, thereby avoiding the increased therapy-related mortality observed in this study. Furthermore, our results serve to reinforce the importance of avoiding therapy with isotonic saline in patients with euvolemic hyponatremia, whatever the cause. Future prospective studies will be needed to confirm the relevance of an adequate and active treatment of euvolemic hyponatremia, albeit mild, for clinical prognosis, not only in the case of COVID-19 patients, but in patients with hyponatremia in general.

## Data availability statement

The raw data supporting the conclusions of this article will be made available by the authors, without undue reservation.

## Ethics statement

The studies involving human participants were reviewed and approved by Research Ethics Committee of the HCSC (20/396-E_COVID). Written informed consent for participation was not required for this study in accordance with the national legislation and the institutional requirements.

## Author contributions

MP-G, JR-S, JM and IR-V contributed to conception and design ofthe study. MP-G and JR-S organized the database and performed the statistical analysis. MP-G, JR-S wrote the first draft of the manuscript. MP-G, JR-S, and IR-V wrote sections of the manuscript. All authors contributed to manuscript revision, read, and approved the submitted version.
